# ^18^FDG-PET at 1-Month Intervals Is a Better Predictive Marker for GISTs That Are Difficult to Be Diagnosed Histopathologically: A Case Report

**DOI:** 10.1155/2011/638794

**Published:** 2011-10-11

**Authors:** Kazunori Otsuka, Masahiro Takahashi, Hiroshi Nanjo, Hideaki Miyazawa, Masatake Iida, Yuki Abe, Mario Jin, Hirohide Onishi, Manabu Hashimoto, Yuzo Yamamoto, Hiroyuki Shibata

**Affiliations:** ^1^Department of Clinical Oncology, Akita University Graduate School of Medicine, 1-1-1 Hondo, Akita 010-8543, Japan; ^2^Division of Clinical Pathology, Faculty of Medicine, Akita University, 1-1-1 Hondo, Akita 010-8543, Japan; ^3^Department of Gastroenterological Surgery, Akita University, 1-1-1 Hondo, Akita 010-8543, Japan; ^4^Department of Gastroenterology and Neurology, Akita University, 1-1-1 Hondo, Akita 010-8543, Japan; ^5^Department of Radiology, Akita University, 1-1-1 Hondo, Akita 010-8543, Japan

## Abstract

Imatinib mesylate is a tyrosine kinase inhibitor of c-KIT and PDGFRA. Imatinib mesylate is an effective drug that can be used as a first-choice agent for treatment of GISTs. Prior to treatment, molecular diagnosis of c-KIT or PDGFRA is necessary; however, in some types of GISTs, it is impossible to obtain a sufficient amount of specimen for diagnosis. An inoperable or marginally resectable GIST in a 79-year-old female was difficult to be diagnosed at a molecular pathological level, and hence, exploratory treatment was initiated using imatinib combined with ^18^FDG-PET evaluation at 1-month intervals. PET imaging indicated a positive response, and so we continued imatinib treatment in an NAC setting for 4 months. As a result, curative resection of the entire tumor was successfully performed with organ preservation and minimally invasive surgery. ^18^FDG-PET evaluation at 1-month intervals is beneficial for GISTs that are difficult to be diagnosed histopathologically.

## 1. Introduction

Gastrointestinal stromal tumors (GISTs) are a type of submucosal tumor (SMT) with a malignant phenotype, derived from interstitial cells of Cajal [1]. It is estimated that the incidence of GISTs is approximately 10 to 20 per million people every year [1]. Oncogenic activation of c-KIT or platelet-derived growth factor receptor *α* (PDGFRA) is responsible for molecular carcinogenesis of GIST [1]. Imatinib mesylate (Ima) is effective for inoperable or metastatic GISTs with permissive toxicities [2–4]. Application of Ima in adjuvant chemotherapy and neoadjuvant chemotherapy (NAC) setting is still under clinical investigations.

 Molecular histpathological analysis is necessary for the diagnosis of GISTs. However, SMTs such as GISTs are often difficult to sample. In this paper, we succeeded in using exploratory treatment combined with ^18^Fluorodeoxyglucose-positron emission computed tomography (^18^FDG-PET) as an alternative method for diagnosing GISTs. 

## 2. Case Presentation

**Figure 1:**
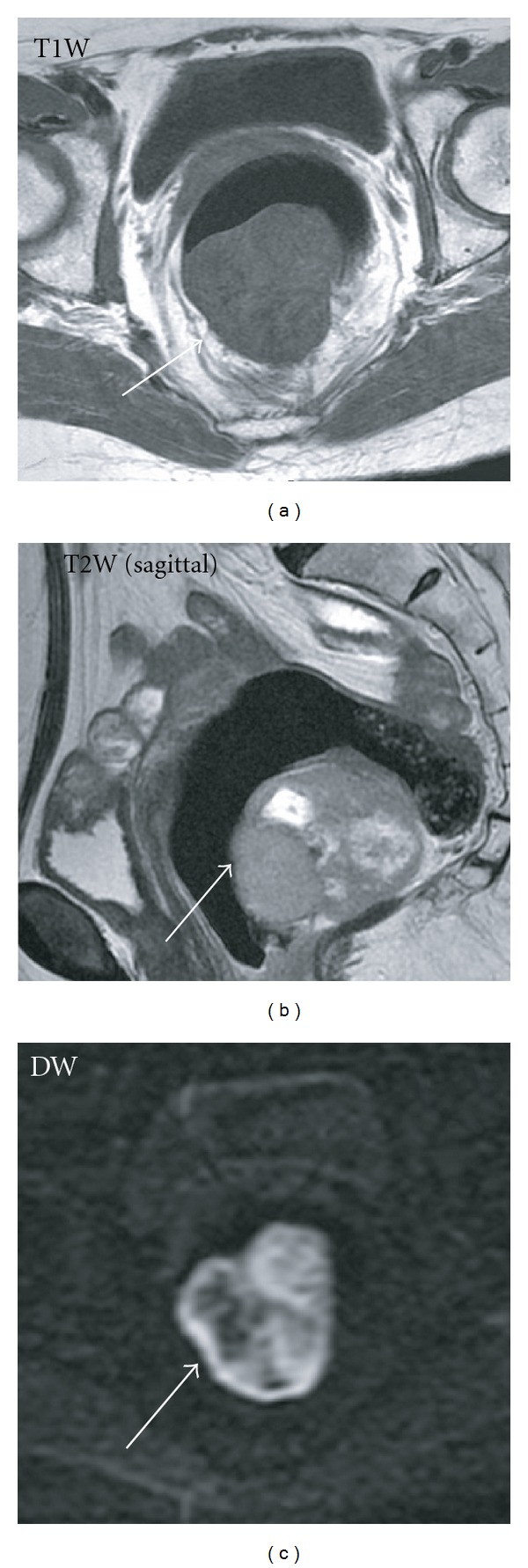
MRI imaging of tumor (T1-weighted (a), T2-weighted (b), and diffusion-weighted (c) images).

**Figure 2:**
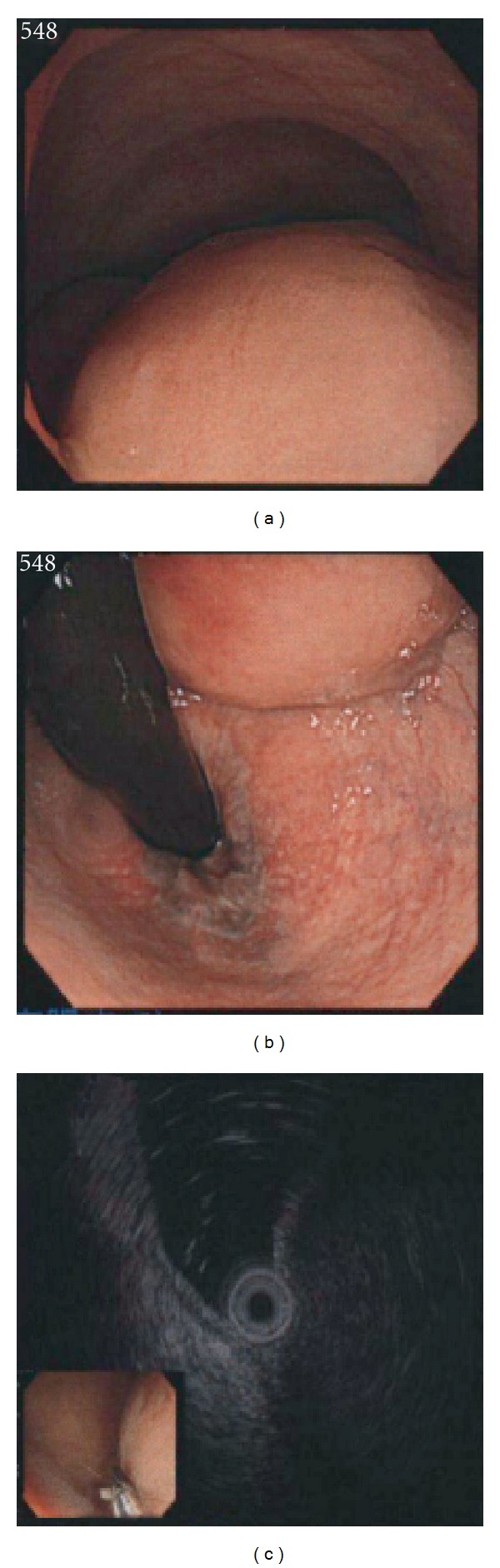
Endoscopic (a, b) and EUS (c) findings.

**Figure 3:**
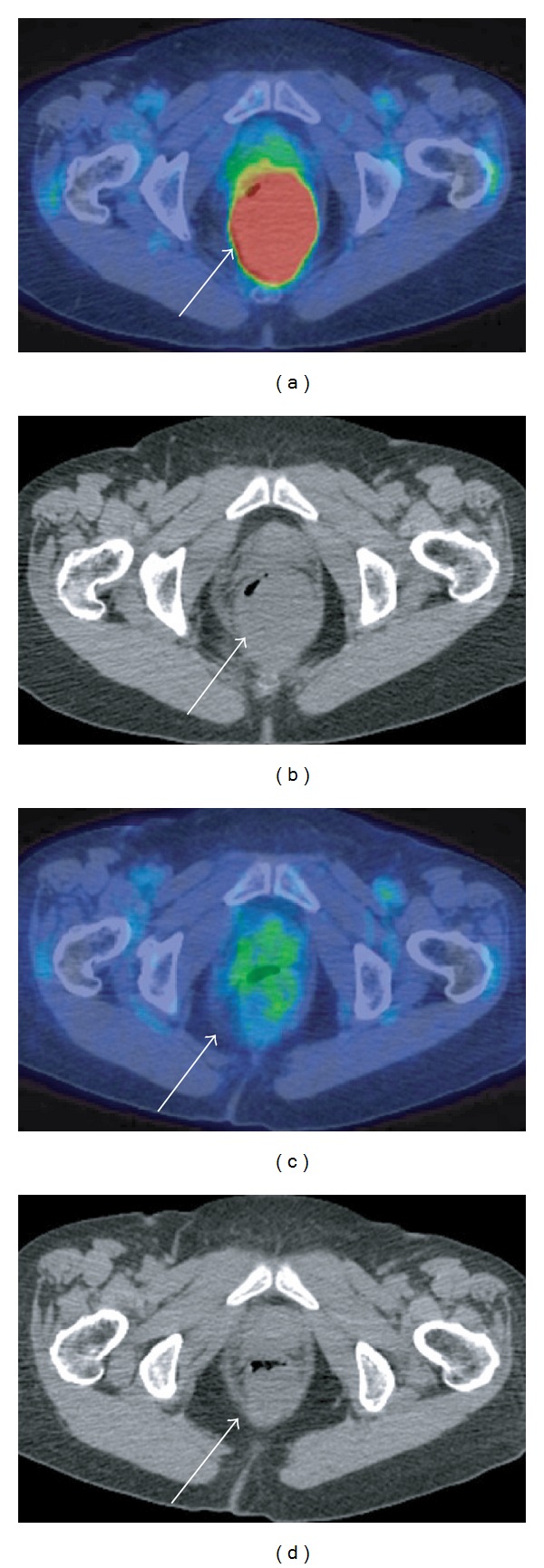
^18^FDG-PET/CT imaging prior to Ima treatment (a, b) and 1 month after treatment (c, d).

**Figure 4:**
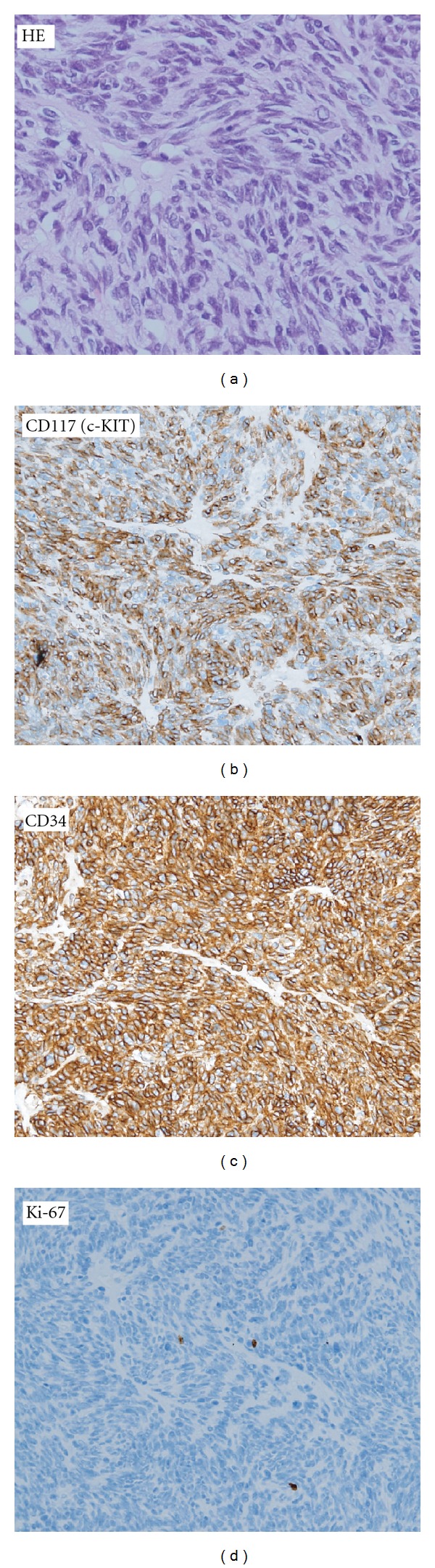
Histopathological analysis of the tumor: (a) HE, (b) CD117, (c) CD34, and (d) Ki-67.

 A 79-year-old Japanese female consulted Akita University Hospital on December 2009 with a history of progressive difficulty with defecation. Abdominal ultrasound (US), abdominal computed tomography (CT) images, and magnetic resonance images (MRIs) revealed a tumor with a maximum diameter of 5.5 cm, occupying the whole cavity of pelvis minor, within the posterior wall of the lower rectum (Figure 1). The T1-weighted image of the tumor showed a low-signal intensity that was identical to that of smooth muscle. Both the T2-weighted and the diffusion-weighted images showed heterogeneous, intermediate-to-high signal intensities in the tumor that were enhanced with intravenous gadolinium chelate. Colonoscopy revealed no mucosal findings except a submucosal mass (Figure 2). Endoscopic US examinations revealed a low-echoic and irregular pattern in the tumor. ^18^FDG-PET showed marked increased uptake in the rectal tumor (Figure 3(a)). The maximal standard uptake value (SUV-max) was 17.4. Results of laboratory workup were almost within normal limits. Judging from this extent, the tumor was not respectable at the expense of rectal amputation, but the risk of iatrogenic tumor rapture and/or injury to the pudendal plexus seemed to be very high. From the risk of bleeding or seeding into the pelvic cavity, biopsy was avoided. Images of the tumor, especially the T2-weighted image, highly suggested the diagnosis of GIST [5]. In this context, initiation of Ima treatment in an NAC setting was worthwhile based on the condition that evaluation of the antitumor effect should be performed after 1 month of treatment. Informed consent was obtained from the patient stating the advantages and disadvantages of the treatment, that is, NAC with Ima. After 5 weeks of administration at a standard dose or less (200–400 mg/day), the antitumor effect was evaluated by ^18^FDG-PET or CT. SUV-max decreased to that of the background level, and the sum of the largest diameter of the tumor to 71% of baseline (Figure 3(c)). The main reasons for dose reduction were anorexia. This tumor was diagnosed as an Ima-sensitive GIST. NAC with Ima was continued for 4 months. Due to the gradually lower compliance of Ima, it was considered that the maximal shrinkage was obtained at this point, and therefore, the patient underwent surgery. Under abdominosacral approach with laparoscopic assistance, complete tumor resection was attained only with a partial resection of the rectal wall. Histopathological analysis of the surgical specimen identified a gray-white elastic soft tumor of 5 cm diameter. No viable cells were observed in two-third portion of the tumor, which presented as liquefactive necrosis or hyaline degeneration. The other part mainly contained spindle-type cells that showed positive staining with CD117 (c-KIT) and CD34, (Figures 4(a), 4(b), and 4(c)). Almost no mitotic figures and very low Ki-67 positivity (0.1%) was observed (Figure 4(d)). Thus, it was concluded that an R0 resection had been performed. Molecular analysis identified a deletion mutation of codon 557/558 in exon 11 of the *c-KIT* gene (557/558 del). Adjuvant chemotherapy with Ima was not performed due to the patient's refusal. After 6 months of followup, there was no evidence of local or distant recurrence.

## 3. Discussion

Inoperable, metastatic, or marginally resectable GISTs are good targets for Ima treatment. Molecular diagnosis confirming c-KIT- or PDGFRA-positive GISTs is a precondition for Ima treatment. However, it is often difficult to obtain a sufficient amount of specimens from SMT-like GISTs. Even with endoscopic ultrasound-guided trucut biopsy, no adequate specimen for diagnosis can be obtained in as many as 37% of gastric SMTs [6]. In 2 out of 52 trucut biopsy procedures, severe septic complications were reported [6]. On the other hand, ^18^FDG-PET is rather harmless and can evaluate the response accurately in 85% of patients at 1 month, whereas CT can evaluate the response in 44% of patients at the same time period [7]. ^18^FDG is simply reflected by elevated glycolysis of cancer cells. ^125^Iodine- and ^111^Indium-labeled anti-c-KIT antibodies are still under investigation [8]. Evaluation by a second ^18^FDG-PET at 1 month is a good surrogate marker of therapeutic response. Thus, exploratory treatment with Ima is permissible, and the issue concerning irradiation was negligible. The average effective dose of a PET scan is 14.1 mSv, that is twice higher than that of a chest CT [9]. However, the maximum annual additional risk for the incidence of cancer is estimated to be 0.019% [9]. Four-month NAC is appropriate against the recent result concerning a phase 2 NAC study of GIST, where Ima (600 mg/day) was continued for 8 to 12 weeks prior to surgery with minimal complications [10]. NAC was proven to be beneficial against a large tumor (>5 cm) and tumors that were potentially morbid on resection from the viewpoint of organ preservation and minimally invasive surgery [11]. The prognosis of GISTs bearing the deletion mutation in exon 11 of the *c-KIT* gene is very poor [12]. However, the therapeutic benefit with Ima was exceptional for a 557/558 del, and it has been shown that progression-free survival is prolonged over 4 years with Ima [13]. 

## References

[1] Stamatakos M, Douzinas E, Stefanaki C (2009). Gastrointestinal stromal tumor. *World Journal of Surgical Oncology*.

[2] Tuveson DA, Willis NA, Jacks T (2001). STI571 inactivation of the gastrointestinal stromal tumor c-KIT oncoprotein: biological and clinical implications. *Oncogene*.

[3] Corless CL, Fletcher JA, Heinrich MC (2004). Biology of gastrointestinal stromal tumors. *Journal of Clinical Oncology*.

[4] Demetri GD, Von Mehren M, Blanke CD (2002). Efficacy and safety of imatinib mesylate in advanced gastrointestinal stromal tumors. *The New England Journal of Medicine*.

[5] Caramella T, Schmidt S, Chevallier P (2005). MR features of gastrointestinal stromal tumors. *Clinical Imaging*.

[6] Polkowski M, Gerke W, Jarosz D (2009). Diagnostic yield and safety of endoscopic ultrasound-guided trucut [corrected] biopsy in patients with gastric submucosal tumors: a prospective study. *Endoscopy*.

[7] Antoch G, Kanja J, Bauer S (2004). Comparison of PET, CT, and dual-modality PET/CT imaging for monitoring of imatinib (STI571) therapy in patients with gastrointestinal stromal tumors. *Journal of Nuclear Medicine*.

[8] Sogawa C, Tsuji AB, Sudo H (2010). C-kit-targeted imaging of gastrointestinal stromal tumor using radiolabeled anti-c-kit monoclonal antibody in a mouse tumor model. *Nuclear Medicine and Biology*.

[9] Devine CE, Mawlawi O (2010). Radiation safety with positron emission tomography and computed tomography. *Seminars in Ultrasound, CT and MRI*.

[10] Eisenberg BL, Harris J, Blanke CD (2009). Phase II trial of neoadjuvant/adjuvant imatinib mesylate (IM) for advanced primary and metastatic/recurrent operable gastrointestinal stromal tumor (GIST): early results of RTOG 0132/ACRIN 6665. *Journal of Surgical Oncology*.

[11] von Mehren M, Watson JC (2009). Perioperative tyrosine kinase inhibitors for GIST: standard or an idea that needs further investigation?. *Oncology*.

[12] Martin-Broto J, Gutierrez A, Garcia-del-Muro X (2010). Prognostic time dependence of deletions affecting codons 557 and/or 558 of KIT gene for relapse-free survival (RFS) in localized GIST: a Spanish Group for Sarcoma Research (GEIS) Study. *Annals of Oncology*.

[13] Blay J, Bin N, Bui PA Correlation of the topography of KIT exon 11 mutation with primary GIST location and predictive value for PFS in patients with advanced GIST: results from the BFR14 andomized phase III trial of the French Sarcoma Group. http://www.asco.org/ASCOv2/Meetings/Abstracts?&vmview=abst_meeting_categories_view&confID=74.

